# From bidirectional pathophysiology to integrative therapeutics: current status and future perspectives of acupuncture for tinnitus-insomnia comorbidity

**DOI:** 10.3389/fpsyt.2025.1688016

**Published:** 2025-12-04

**Authors:** Hantong Hu, Xiaohan Huang, Yang Wang, Yang Li, Lianqiang Fang, Jie Zhou, Hong Gao

**Affiliations:** 1 Department of Acupuncture and Moxibustion, The Third Affiliated Hospital of Zhejiang Chinese Medical University, Hangzhou, China; 2 Department of Neurobiology and Acupuncture Research, Zhejiang Chinese Medical University, Key Laboratory of Acupuncture and Neurology of Zhejiang Province, Hangzhou, China; 3 The Third Clinical College, Zhejiang Chinese Medical University, Hangzhou, Zhejiang, China

**Keywords:** tinnitus, insomnia, acupuncture, comorbidity, bidirectional relationship, neuroplasticity

## Abstract

This perspective article discusses the complex bidirectional relationship between tinnitus and insomnia, a prevalent and burdensome comorbidity. It synthesizes evidence suggesting the conditions mutually exacerbate one another through shared pathophysiological mechanisms, including central hyperarousal, emotional dysregulation, and aberrant neural network connectivity. Our study then explores acupuncture as a potential integrated therapy. Preliminary clinical studies indicate acupuncture can concurrently alleviate both tinnitus and insomnia symptoms. Its therapeutic potential is attributed to multi-target effects, such as regulating the autonomic nervous system and HPA axis, modulating neurotransmitters and neuroinflammation, and normalizing functional brain networks. Our study concludes by highlighting the critical need for rigorous research focusing specifically on the comorbidity to validate these mechanisms and establish clinical efficacy.

## 1 Introduction

Tinnitus refers to the perception of sound in the ear or head without external sound sources. The causes of tinnitus are diverse, including hearing loss, noise exposure, ear infections, vascular abnormalities, and side effects of certain medications. Tinnitus has a prevalence of ~14% in the general population and is expected to become increasingly prevalent, posing a growing health burden (1). This burden extends beyond the mere perception of sound to impair critical functions such as speech comprehension, even in cases with normal pure-tone audiometry results, highlighting its central nervous system components (2). On the other hand, insomnia is one of the most common sleep disorders, characterized primarily by difficulty falling asleep, maintaining sleep, or early awakening. According to a recent meta-analysis, insomnia is a common disorder with a prevalence of 12.4 as the most accurate estimate in the general population (3). Chronic insomnia not only affects daytime cognitive function and emotional state but may also increase the risk of chronic diseases such as cardiovascular disease and diabetes.

Notably, complex interactive relationships exist between tinnitus and insomnia, and tinnitus-insomnia comorbidity is very common in clinical practice (4). This comorbid phenomenon not only increases diagnostic and therapeutic complexity but also brings dual suffering and burden to patients. Based on epidemiological reports, the proportion of tinnitus patients seeking sleep treatment ranges from 50% to 77% (5). This high proportion indicates the universality and severity of tinnitus’s impact on sleep. In recent years, increasing research has focused on the bidirectional relationship between tinnitus and insomnia, attempting to reveal how tinnitus affects sleep and the possible mechanisms by which insomnia affects tinnitus. However, current understanding of the deep mechanisms underlying this bidirectional interactive relationship remains limited, particularly with obvious deficiencies in finding effective comprehensive treatment strategies.

This perspective article will analyze relevant literature to explore the potential mechanisms of mutual influence between tinnitus and insomnia, and further discuss the clinical efficacy and potential mechanisms of acupuncture therapy in treating tinnitus-insomnia comorbidity. Of note, It is important to acknowledge that the pathophysiological mechanisms of both tinnitus and insomnia individually remain incompletely understood, making the study of their comorbidity even more challenging. Furthermore, research specifically investigating acupuncture mechanisms for this particular comorbidity is virtually nonexistent. Therefore, this perspective article aims not to provide definitive mechanistic conclusions, but rather to synthesize current fragmentary evidence, identify critical knowledge gaps, propose testable hypotheses, and define future research directions for this understudied clinical entity.

## 2 Bidirectional relationship between tinnitus and insomnia

### 2.1 Mechanisms by which tinnitus affects insomnia

Tinnitus is one of the common causes of insomnia (6). Chronic tinnitus causes persistent sleep disturbances, and even when tinnitus is partially alleviated, sleep problems may continue to persist. Tinnitus could affect sleep through the following mechanisms.

#### 2.1.1 Auditory interference

Tinnitus generates internal noise that directly disrupts the process of falling asleep, increases nocturnal awakenings, and ultimately reduces sleep quality (6). This form of sensory interference is particularly pronounced in quiet nighttime environments, where ambient masking sounds are absent, thereby making the internal noise more perceptible and intrusive. The auditory interference mechanism operates through multiple pathways: the phantom sound perception continuously activates the ascending reticular activating system (ARAS), maintaining cortical arousal and preventing the transition to sleep stages (7). Furthermore, the persistent auditory input competes with the brain’s natural tendency to reduce sensory processing during sleep initiation, creating a state of sustained vigilance. This interference not only delays sleep onset but also fragments sleep architecture by triggering micro-arousals throughout the night (8).

#### 2.1.2 Attention focus

Tinnitus tends to become more noticeable at night due to reduced environmental noise, thereby drawing the patient’s attention and interfering with relaxation and sleep onset. This heightened attentional focus can lead to a vicious cycle—efforts to ignore the tinnitus paradoxically result in increased attention to it, thereby exacerbating sleep disturbance.

#### 2.1.3 Mediation by negative emotions

Tinnitus often triggers negative emotional responses such as frustration, anxiety, and depression, which indirectly impair sleep. Studies have shown that patients with chronic tinnitus experience greater sleep-related worry and emotional distress compared to healthy controls (9). Anxiety and depression are common comorbidities of tinnitus and are also key risk factors for insomnia, thereby establishing a strong emotional bridge between tinnitus and insomnia. Indeed, many studies have investigated this relationship and found a significant positive correlation between the Tinnitus Handicap Inventory (THI) and scores for depression and anxiety, thereby suggesting the tinnitus handicap has a profound effect on the manifestation of these psychiatric symptoms (10).

#### 2.1.4 Cognitive-behavioral maintenance

Some research suggests that insomnia resulting from tinnitus may be maintained through cognitive-behavioral processes similar to those seen in primary insomnia. Patients with tinnitus-related insomnia may benefit from cognitive-behavioral therapy for insomnia (CBT-I) (9). Maladaptive cognitive patterns—such as excessive worry about sleep, catastrophic thinking, and dysfunctional beliefs about tinnitus—could collectively maintain and exacerbate insomnia symptoms.

### 2.2 Mechanisms by which insomnia affects tinnitus

Corresponding to tinnitus affecting sleep, insomnia can exacerbate tinnitus through the following mechanisms, forming a bidirectional vicious cycle.

First, insomnia leads to increased brain sensitivity to noise, making originally mild tinnitus perception more intense. Insomnia is believed to worsen tinnitus loudness and severity, with research finding that insomnia patients report significantly higher tinnitus loudness and severity compared to tinnitus patients without insomnia problems (5). This heightened sensitivity may be linked to functional abnormalities in the central auditory processing system caused by sleep deprivation.

Second, insomnia exacerbates anxiety and depression, thereby intensifying the impact of negative emotions on tinnitus (10). Sleep deprivation reduces individuals’ emotional regulation capacity, making it more difficult for patients to cope with tinnitus-related distress.

Third, insomnia may lead tinnitus patients to focus attention on sound symptoms, exacerbating subjective perception. Sleep deprivation affects attention allocation and control, making it more difficult for patients to shift attention from tinnitus to other activities or sensations.

### 2.3 Mechanisms underlying the bidirectional relationship between tinnitus and insomnia

#### 2.3.1 Emotional regulation: bidirectional mediation role of negative emotions in tinnitus and insomnia

Emotional regulation mechanisms play a key role in the pathological process of tinnitus-insomnia comorbidity. Research has found that persistent noise and discomfort from tinnitus easily trigger negative emotions such as anxiety and depression, and these negative emotions in turn affect sleep quality. Negative emotions such as anxiety and depression play important mediating roles in the bidirectional effects of insomnia and tinnitus (10). This emotional mediation mechanism involves not only excessive activation of the limbic system but also impairment of prefrontal cortex emotional regulation function.

#### 2.3.2 Hyperarousal: shared pathway through sympathetic nervous system activation

Hyperarousal is another core pathophysiological mechanism. Some studies propose that tinnitus and insomnia share overlapping mechanisms, one of which is heightened sympathetic nervous system activity leading to a persistent state of hyperarousal (11). Both tinnitus and insomnia are frequently accompanied by increased sympathetic tone, which may underlie their mutual exacerbation. Hyperarousal manifests not only as enhanced sympathetic nervous activity at the physiological level but also includes increased alertness at the cognitive level and increased irritability at the emotional level.

#### 2.3.3 Neural network abnormalities: brain region basis for tinnitus-insomnia interaction

Neural network connectivity abnormalities constitute the neuroanatomical basis for the interactive effects of insomnia and tinnitus. The bidirectional relationship between tinnitus and insomnia may involve interactions between multiple neural pathways and brain regions. Research has found that tinnitus patients have abnormal neural functional connections between auditory centers and non-auditory centers (such as frontal lobe, temporal lobe, limbic system) (12). Insomnia may exacerbate tinnitus symptoms by affecting these neural pathways and brain regions. Functional abnormalities in these brain regions not only affect auditory information processing but also involve integration of multiple functional domains including attention, emotional regulation, and sleep-wake modulation.

#### 2.3.4 Central nervous plasticity: shared neural mechanism regulating tinnitus and sleep disorders

Central plasticity may play a bridging role in the interactive effects of tinnitus and insomnia (13). The pathogenesis of both tinnitus and insomnia is closely related to neuroplasticity changes in the cerebral cortex. These plasticity changes include regulation of synaptic connection strength, changes in neuronal excitability, and neural network reorganization at multiple levels, which may simultaneously affect neural circuits for auditory processing and sleep regulation. Furthermore, systemic alterations, such as elevated fasting glucose and blood lipid levels, may impact central processing and contribute to the pathology of tinnitus (14). Therefore, these systemic metabolic parameters warrant consideration as potential mediators of both tinnitus and insomnia comorbidity, as metabolic dysfunction may compromise neuroplasticity.

#### 2.3.5 Hierarchical interactions and potential core drivers in the comorbid state

While the mechanisms described above (emotional dysregulation, hyperarousal, neural network abnormalities, and neuroplasticity) clearly contribute to the bidirectional relationship between tinnitus and insomnia, their hierarchical organization and potential interactions remain poorly understood. To conceptualize these relationships, we propose a classification framework based on temporal dynamics and mechanistic roles: (1) precipitating factors that initiate the comorbid state, (2) perpetuating mechanisms that maintain chronicity, and (3) integrative drivers that coordinate multiple pathophysiological processes. This classification draws upon established models in chronic disease comorbidity research, which emphasize distinguishing between initiating triggers and maintenance mechanisms (15).

We hypothesize that central hyperarousal serve as a critical integrative driver that both results from and perpetuates the other pathophysiological processes. This hypothesis is supported by hyperarousal models in comorbid psychiatric and somatic conditions, which position hyperarousal as a transdiagnostic mechanism linking multiple symptom domains (11, 16). Specifically, emotional dysregulation and negative emotions may may serve as precipitating factors that trigger and establish hyperarousal states; hyperarousal then functions as a perpetuating mechanism that amplifies attentional focus on tinnitus and prevents sleep initiation; chronic sleep deprivation further dysregulates emotional processing; and sustained hyperarousal drives maladaptive neuroplastic changes in relevant neural networks (11).

Alternatively, emotional dysregulation may represent a central mediating pathway functioning as an integrative driver, as supported by evidence showing that anxiety and depression mediate the relationship between tinnitus severity and sleep disturbance (10). Under this framework, both tinnitus and insomnia would serve as precipitating stressors that independently trigger negative emotional responses, which then function as perpetuating mechanisms that reciprocally exacerbate both conditions.

In addition, from a neurobiological perspective, neural network abnormalities constitute the fundamental substrate upon which other mechanisms operate. Aberrant connectivity patterns in key brain networks may represent underlying vulnerability factors (precipitating mechanisms) that predispose individuals to both conditions (12, 13), with hyperarousal and emotional dysregulation emerging as secondary perpetuating consequences (10, 11).

These competing hypotheses highlight the need for future research employing advanced statistical modeling (e.g., mediation analyses, network analysis) to map the temporal and causal relationships among these mechanisms in the comorbid state (5, 15). Such investigations should examine whether certain mechanisms show primacy in symptom onset versus maintenance phases, and whether individual differences (e.g., tinnitus subtype, insomnia phenotype, psychological profiles) influence which pathways predominate.

#### 2.3.6 Summary of bidirectional mechanisms

In summary, the bidirectional relationship between tinnitus and insomnia appears to be mediated by multiple interconnected mechanisms operating at different hierarchical levels. While we have identified four primary pathophysiological domains (i.e., emotional dysregulation, central hyperarousal, neural network abnormalities, and maladaptive neuroplasticit), the precise hierarchical organization and causal primacy of these mechanisms remain to be definitively established. Importantly, these mechanisms likely do not operate in isolation but rather form an interactive network in which perturbations in one domain cascade through others, creating the self-perpetuating cycle characteristic of chronic comorbidity (15). Unveiling these complex hierarchical relationships in future research has direct clinical implications: identifying core drivers could enable more targeted therapeutic interventions that address root causes rather than superficial symptoms. The integrative nature of acupuncture therapy, which simultaneously modulates multiple physiological systems may be particularly well-suited to address this multi-mechanistic comorbidity.

## 3 Acupuncture treatment of tinnitus-insomnia comorbidity

### 3.1 Clinical research on acupuncture treatment of tinnitus/insomnia comorbidity

Jackson et al. (17) explored the effect of acupuncture on tinnitus and insomnia. In their study, six patients with tinnitus underwent two weeks of acupuncture therapy, resulting in significant reductions in the loudness and pitch of tinnitus (both rated 0-10, where 10 the worst possible), along with notable improvements in sleep quality (rated 0-10, where 10 the worst possible). Although the sample size was limited, this study provided preliminary clinical evidence supporting the use of acupuncture for coexisting tinnitus and insomnia. In another clinical trial by Laureano et al., acupuncture was found to improve anxiety and depression scores in tinnitus patients (18).

In China, a randomized controlled trial (RCT) (19) showed that acupuncture (three sessions weekly for four weeks) led to significant improvements in both tinnitus and insomnia symptoms among patients with both conditions by the end of the four-week treatment period. Notably, insomnia severity index (ISI) scores improved significantly after treatment. Another RCT (20) compared acupuncture to pharmacological treatment [acupuncture treatment protocol: once daily for 4 weeks; pharmacological treatment: estazolam 1 mg nightly for 4 weeks] in patients with neurogenic tinnitus comorbid with insomnia found superior outcomes in the acupuncture group. At the end of the 4-week treatment, the overall effective rates for tinnitus and insomnia in the acupuncture group were 52.94% and 58.82%, respectively, which were significantly higher than those in the control group receiving Estazolam (38.71% and 41.94%). These findings support the clinical value of acupuncture in managing comorbid tinnitus and insomnia. However, long-term follow-up data extending beyond the 4-week treatment period were not reported in the original studies (19, 20), leaving uncertainty regarding whether the observed improvements were sustained or whether additional long-term therapeutic effects were present. Furthermore, a recent systematic review and meta-analysis also concluded that acupuncture significantly alleviates tinnitus-related insomnia, anxiety, and depressive symptoms (21).

Collectively, these studies provide meaningful clinical and evidence-based insights into the dual therapeutic potential of acupuncture in treating tinnitus-insomnia comorbidity.

#### 3.1.1 Critical appraisal of clinical evidence

Despite these encouraging preliminary findings, it is essential to critically evaluate the limitations of existing clinical evidence. First, sample sizes have been notably small (e.g., Jackson et al. included only 6 patients), substantially limiting statistical power and generalizability. Second, methodological quality varies considerably: many studies lack adequate randomization procedures, blinding of outcome assessors, or appropriate placebo/sham controls—factors that introduce significant risk of bias. Third, outcome measures have relied predominantly on subjective self-reports without objective verification (e.g., polysomnography for sleep, psychoacoustic measures for tinnitus). Fourth, follow-up periods have been short (typically 4 weeks or less), precluding assessment of long-term efficacy and durability of treatment effects. Fifth, none of the identified studies employed standardized, validated protocols specifically designed for the comorbidity, limiting reproducibility and cross-study comparison. Sixth, potential confounding factors such as concurrent medications, comorbid psychiatric conditions, and tinnitus etiology have not been systematically controlled or analyzed. These methodological limitations substantially reduce the certainty of evidence and underscore that current findings, while promising, must be considered preliminary and hypothesis-generating rather than conclusive. Definitive conclusions regarding acupuncture’s efficacy for tinnitus-insomnia comorbidity await well-designed, adequately powered, rigorously controlled randomized clinical trials.

#### 3.1.2 Proposed clinical protocol for tinnitus-insomnia comorbidity

While a standardized protocol for treating tinnitus-insomnia comorbidity with acupuncture has yet to be established, evidence from existing studies (17–20) can inform a preliminary clinical protocol for tinnitus-insomnia comorbidity. This proposed protocol serves as a reference for clinical practice and a foundation for designing rigorous clinical trials to validate the efficacy of acupuncture for this specific comorbidity.

##### 3.1.2.1 Acupuncture modalities

Manual acupuncture (MA) and electroacupuncture (EA) represent the most extensively studied approaches, with EA showing advantages in modulating neural network connectivity and neurotransmitter regulation. Auricular acupuncture has also emerged as a valuable adjunctive therapy, particularly for its accessibility and sustained stimulation effects.

##### 3.1.2.2 Acupoint selection

A potential protocol would involve a combination of local points around the ear, distal points on the limbs, and acupoints known for calming the mind. A core acupoint prescription could generally include:

- Local points, such as TE17 (Yifeng), SI19 (Tinggong), GB2 (Tinghui), to directly target the auditory system and improve local circulation.
- Distal points for tinnitus based on meridian theory, such as TE3 (Zhongzhu), LI4 (Hegu).
- Points for insomnia and emotional regulation, such as HT7 (Shenmen), PC6 (Neiguan), SP6 (Sanyinjiao), to calm the spirit, regulate the hypothalamic-pituitary-adrenal (HPA) axis, and improve sleep quality.

##### 3.1.2.3 Treatment course

A typical treatment course generally involves 2–3 sessions per week for a total of 4–8 weeks. The duration and frequency could be adjusted based on the patient’s treatment response. Each treatment session of acupuncture generally last 30 minutes.

### 3.2 Potential mechanisms of acupuncture in treating tinnitus-insomnia comorbidity

The following mechanistic discussion presents evidence-based hypotheses regarding how acupuncture might address tinnitus-insomnia comorbidity. It is critical to emphasize that these represent theoretical propositions requiring empirical validation rather than established mechanisms. Our approach employs triangulation of evidence from three sources: (1) documented effects of acupuncture on specific physiological systems and mechanisms; (2) established roles of these systems/mechanisms in the pathophysiology of tinnitus and/or insomnia individually; and (3) preliminary clinical observations of acupuncture’s dual efficacy. While direct mechanistic studies in comorbid populations are lacking, this triangulated evidence provides a rational basis for hypothesis generation and can guide future mechanistic research. Each proposed mechanism below should be interpreted as a testable hypothesis rather than a confirmed pathway.

The mechanisms through which acupuncture treats tinnitus involve several aspects. At the central level, acupuncture may reduce tinnitus symptoms by modulating auditory cortex function and the connectivity of related brain networks, such as the default mode network and salience network (22). Peripherally, it may improve cochlear blood flow (23), while centrally enhancing cerebral oxygenation through increased regional oxyhemoglobin concentrations (24). Acupuncture also helps balance sympathetic and parasympathetic activity, thus reducing associated symptoms such as anxiety and stress (25). These multifaceted actions suggest that acupuncture exerts broad neuromodulatory, circulatory, and autonomic regulatory effects on tinnitus.

In the treatment of insomnia, acupuncture’s mechanisms are relatively well-characterized. These include modulation of neurotransmitters such as norepinephrine, melatonin, gamma-aminobutyric acid (GABA), and endogenous opioids to improve sleep quality (26), along with autonomic rebalancing by reducing sympathetic activity and enhancing parasympathetic tone (27). Acupuncture may also improve sleep architecture by decreasing light sleep, increasing deep and REM sleep durations (28), and exert anti-inflammatory and antioxidant effects that further alleviate insomnia symptoms (29). These effects highlight the comprehensive physiological and neurochemical regulation achieved through acupuncture.

Despite the clinical promise, mechanistic studies specifically addressing acupuncture’s role in treating the comorbidity of tinnitus and insomnia are still sparse. Nevertheless, based on existing theories and indirect evidence, several potential pathways can be postulated.

#### 3.2.1 Modulation of emotional states through “regulating the spirit”

Anxiety and depression have been identified as mediators of the bidirectional relationship between tinnitus and insomnia (10). Acupuncture may alleviate these emotional disturbances by “regulating the spirit,” a core concept in traditional Chinese medicine referring to the harmonization of mental and emotional states. This regulation may involve rebalancing neurotransmitters, modulating neuroendocrine axes, and enhancing functional connectivity in emotion-related brain regions (30). Modern neuroscience research supports these effects; for example, acupuncture has been shown to activate the prefrontal cortex and modulate limbic system activity, thereby improving emotional regulation.

#### 3.2.2 Modulation of gut microbiota dysbiosis

Emerging evidence links disturbances in the gut microbiome to both tinnitus and insomnia. For example. Megantara et al. reviewed the role of gut microbiota imbalance in tinnitus pathogenesis, emphasizing its impact on neurotransmitter synthesis and neuroinflammation (31). Additional studies have reported bidirectional interactions between gut flora and sleep, indicating that microbial composition can influence sleep quality via the gut-brain axis (32). Acupuncture has been shown to effectively regulate gut microbiota, enhancing microbial diversity, increasing beneficial bacteria, and reducing pro-inflammatory species. Through this gut-brain regulatory mechanism, acupuncture may alleviate both tinnitus and insomnia by attenuating neuroinflammatory responses and optimizing neurochemical balance.

#### 3.2.3 Regulation of neurotransmitter balance

Both tinnitus and insomnia are associated with dysregulation of multiple neurotransmitters, including GABA, serotonin, dopamine, and norepinephrine. Acupuncture may modulate these neurotransmitters via multiple mechanisms—enhancing synthesis, promoting release, or inhibiting reuptake. For instance, acupuncture has been reported to activate GABAergic neurons, suppressing excessive neuronal excitability (33), which could reduce the perceptual intensity of tinnitus and simultaneously promote sleep. Additionally, acupuncture-induced modulation of the serotonergic system may improve mood (34), indirectly mitigating symptoms of both tinnitus and insomnia.

#### 3.2.4 Modulation of functional brain network connectivity

Recent neuroimaging studies have identified abnormal connectivity patterns in key brain networks among patients with tinnitus and insomnia (35, 36). Acupuncture may normalize the connectivity of these networks—such as the default mode network, salience network, and central executive network—thereby alleviating both conditions (37). Specifically, restoring functional connectivity between the auditory cortex and prefrontal cortex may relieve tinnitus, while modulating activity in sleep-related regions such as the hypothalamus and brainstem may enhance sleep quality. This network-level modulation reflects the systemic and integrative therapeutic nature of acupuncture.

#### 3.2.5 Modulation of the HPA axis

Hyperactivity of the HPA axis plays a critical role in the pathophysiology of both tinnitus and insomnia (38, 39). Chronic stress-induced overactivation of the HPA axis not only intensifies the subjective perception of tinnitus but also disrupts normal sleep-wake rhythms. Acupuncture may simultaneously improve tinnitus and insomnia by regulating HPA axis activity, reducing cortisol levels, and simultaneously regulating melatonin secretion rhythms (40).

#### 3.2.6 Anti-inflammatory and antioxidant effects

Chronic inflammation and oxidative stress play important roles in the pathogenesis of both tinnitus and insomnia. Excessive production of inflammatory factors such as Tumor Necrosis Factor-alpha (TNF-α), Interleukin-1β (IL-1β), and Interleukin-6 (IL-6) not only damages inner ear structures, causing or exacerbating tinnitus (41), but also interferes with sleep regulation mechanisms. Acupuncture has significant anti-inflammatory and antioxidant effects, improving inflammatory microenvironments by activating endogenous antioxidant systems, inhibiting pro-inflammatory factor production, and promoting anti-inflammatory factor release (42). These anti-inflammatory and antioxidant effects provide an immunoregulatory mechanistic basis for acupuncture’s dual benefits in alleviating both tinnitus and insomnia.

#### 3.2.7 Regulation of autonomic nervous system balance

Dysregulation of the autonomic nervous system represents a common pathophysiological underpinning of both tinnitus and insomnia. Increased sympathetic activity and insufficient parasympathetic tone not only enhance the perceptual severity of tinnitus but also contribute to a sustained hyperarousal state that impairs sleep quality. Acupuncture has been shown to modulate autonomic function by stimulating specific acupoints to suppress sympathetic activity while enhancing parasympathetic tone. This rebalancing creates a physiological environment conducive to symptom relief in both conditions (43). The bidirectional regulation of the autonomic nervous system underscores acupuncture’s capacity to address the shared pathogenesis of tinnitus and insomnia simultaneously.

## 4 Discussion

Tinnitus and insomnia exhibit a complex and reciprocal relationship, functioning both as causes and consequences of one another through shared and overlapping mechanisms, including systemic biological factors such as inflammation and hematological parameters (44). This bidirectional interaction suggests the need for integrated treatment approaches rather than isolated symptom management. A deeper understanding of the interplay between tinnitus and insomnia is essential for the development of effective therapeutic and interventional strategies.

As a cornerstone of traditional Chinese medicine, acupuncture—including various modalities such as manual acupuncture, electroacupuncture, and auricular acupuncture—is widely used in clinical practice to manage tinnitus (45) and insomnia (46). Its therapeutic advantages lie in multi-target and multi-level regulation, which aligns closely with the multifaceted pathophysiology of tinnitus-insomnia comorbidity. Recent advances have further elucidated acupuncture’s mechanisms in treating subjective tinnitus, including enhancement of inner ear microcirculation through regulation of vertebrobasilar artery blood supply, reduction of immuno-inflammatory factors to protect cochlear hair cells, modulation of 5-hydroxytryptamine receptors in the cochlear nucleus, and functional reorganization of the auditory cortex and synaptic networks (47). However, most existing clinical studies and systematic reviews/meta-analyses focus exclusively on acupuncture’s effects on either tinnitus or insomnia in isolation (21, 48, 49), overlooking the potential integrative benefits of treating both conditions concurrently. This limited perspective may underestimate acupuncture’s full therapeutic potential and hinder the development of optimized clinical protocols.

Given the frequent co-occurrence and mutual reinforcement of tinnitus and insomnia, understanding whether acupuncture can address both conditions simultaneously is a critical clinical question. To date, studies explicitly investigating this comorbidity remain scarce, representing a significant gap in the field (50). This research void hampers both mechanistic exploration and clinical advancement. Although preliminary clinical findings suggest that acupuncture may improve both symptoms, the underlying mechanisms remain insufficiently defined. Moreover, existing studies often suffer from small sample sizes, suboptimal study design, and limited outcome measures, reducing their capacity to generate high-quality evidence.

In light of these limitations, a more concrete and feasible research pathway is required. The immediate priority should be to first confirm the clinical efficacy of acupuncture for this specific comorbidity through well-designed, large-scale randomized controlled trials (RCTs). These trials must adhere to the gold standard, employing rigorous blinding, appropriate control groups (e.g., sham acupuncture), standardized and validated protocols, and both subjective (e.g., THI, ISI) and objective (e.g., polysomnography) outcome measures. Long-term follow-up is essential to assess the durability and safety of the treatment. Subsequently, techniques such as neuroimaging (e.g., fMRI) and molecular biology (e.g., biomarker analysis) could be used to retrospectively explore the underlying mechanisms in the cohort of patients who have demonstrated a clear clinical response. This approach, which roots mechanistic exploration in confirmed clinical efficacy, is more rigorous than starting from speculative mechanisms. While our perspective article provides a broad overview of plausible mechanisms, we acknowledge that future perspective articles could also benefit from a deeper, more focused exploration of one or two of the most plausible pathways, such as central hyperarousal and autonomic nervous system balance, to formulate more specific, testable hypotheses.

In practical terms, identifying the precise targets and pathways of acupuncture will enhance its clinical precision and effectiveness, ultimately enabling more tailored treatment strategies for patients with tinnitus-insomnia comorbidity. Future investigations should also address individual differences in treatment response based on tinnitus subtype or insomnia phenotype, providing the groundwork for personalized medicine.

### 4.1 Limitations

Several important limitations of this perspective must be acknowledged. First, as a hypothesis-generating work addressing an emerging field with limited direct evidence, many of our mechanistic propositions remain speculative and unvalidated. The triangulation approach we employed (synthesizing evidence from acupuncture studies in other conditions, pathophysiology studies of individual conditions, and preliminary clinical observations) provides rational hypotheses but cannot substitute for direct mechanistic investigation in comorbid populations. Second, the clinical evidence base we reviewed suffers from significant methodological limitations including small sample sizes, risk of bias, heterogeneous protocols, and short follow-up periods. Third, we proposed a preliminary clinical protocol that, while grounded in theory and available evidence, has not been formally validated or optimized through systematic research. Fourth, we could not definitively establish hierarchical relationships among the multiple bidirectional mechanisms discussed, as current evidence does not permit such determination. These limitations underscore that our work should be interpreted as defining a research agenda and proposing testable hypotheses rather than providing definitive conclusions.

## 5 Conclusions

In summary, although research on acupuncture treatment for tinnitus/insomnia comorbidity is still in its early stages, both its theoretical foundation and preliminary clinical evidence show good application prospects. With the deep development of multidisciplinary cross-research and widespread application of modern technological methods, we have reason to believe that acupuncture will play a greater role in treating tinnitus/insomnia comorbidity, bringing better treatment effects and quality of life improvements to patients. This not only benefits the modern development of traditional medicine but also provides new therapeutic ideas and methods for modern medicine.
